# Hand Hygiene Messaging Design in the Workplace: Views From the Workforce—Introduction

**DOI:** 10.1177/19375867231195646

**Published:** 2023-09-20

**Authors:** Catherine Stones, Wenbo Ai, Sophie Rutter, Andrew Madden

**Affiliations:** 1 School of Design, University of Leeds, United Kingdom; 2 Royal College of Art, London, United Kingdom; 3 Information School, Sheffield University, London, United Kingdom

**Keywords:** handwashing, research-informed design, participatory design, communication, graphic design

## Abstract

**Aims:**

The study aimed to (1) discover workers’ attitudes toward the use of novel video screens to improve hand sanitization in the workplace and (2) discover what workers’ preferences are for hand hygiene (HH) messaging style and tone and reasons for their preferences.

**Background:**

Practicing good HH in non-medical office settings is vital to curb the spread of a range of common and infectious diseases. Despite this, workers are rarely consulting in the construction of HH messages. The qualitative views of users can provide us with the “why” rather than the “what” and can highlight areas of cynicism, concern and overall attitudes to HH.

**Methods:**

A survey was completed by 520 UK workers concerning attitudes and views toward HH messaging and the use of a video-based hand sanitizer unit. Analysis consisted of both qualitative and quantitative methods.

**Results:**

Workers were skeptical toward the use of digital technologies within HH interventions, and there were misgivings about the role that video could play. Results demonstrated a strong preference for positive and supportive messages. Educational and trustworthy qualities were well rated. Messages that emphasized surveillance, previously successful in a clinical setting, or guilt, were not well received. Visual approaches that utilized serious illustration were valued.

**Conclusion:**

This study highlights how consulting workers before the design of HH initiatives is important in guiding the design process. The resultant user-centered criteria promotes the use of positive, motivational, thought-provoking, surprising, and visual approaches to HH messaging.

## Introduction

Practicing good hand hygiene (HH) in office settings is vital to curb the spread of a range of common and infectious diseases (Zivich et al., 2018). For example, Huber et al.’s (2010) study showed how use of hand sanitizer in the workplace significantly reduced the number of episodes of common cold, fever, and coughing. Since the outbreak of COVID-19, hand sanitizer stations have been recommended, featuring in health and safety (H&S) guidance from public health organizations such as the Centers for Disease Control and Prevention (CDC) and Public Health England (PHE). The rapid spread of hand sanitizers (Berardi, 2020) has also resulted in emerging new technologies in the office environment (Courtney, 2021), such as video screens and real-time messaging. These new technologies invite new HH messaging strategies as workers are presented with dynamic displays and interaction capabilities. How we design messages for technology-driven devices in the workplace is the key concern of this paper.

## Aims

The aim of this study was to gauge UK office workers’ attitudes toward a new hand sanitizer station that featured a responsive video screen, in particular, to gather views on the use of messaging to improve HH in the workplace. The study aimed to discover (a) workers’ attitudes toward the use of novel video screens to improve hand sanitization in the workplace and (b) workers’ preferences in terms of HH messaging style and tone and why.

## Significance

This work is significant for a number of reasons. First, gauging attitudes to messaging strategies, while initially seen as subservient to measuring actual HH behavior, still offers high value (Jenner et al., 2005; Lambe et al.,2021). The findings highlighted here are useful for any researcher at the early stage of HH intervention planning, as a guide for strategies to test or avoid.

Second, this study fills a clearly identified gap in the literature. Studies examining attitudes toward HH messaging or campaigns are few in number and focus on clinical settings (Ghanbari, 2014). It is imperative that work is extended into the workplace. While the notion of patient empowerment related to HH is well established (Pittet et al., 2000), there are opportunities in the way general office workers are consulted about HH initiatives.

Third, while the focus of this study was on capturing ideas and responses to the novel integration of a video screen within a hand sanitizer unit, findings are very relevant to broader applications in signage and poster-led approaches to HH messaging.

## Theoretical and Academic Context

HH interventions formulated and evaluated in the academic domain are informed by models and frameworks of behavior change, for example, The Health Belief Model (Ghanbari, 2014), Theoretical Domain Framework (Fuller et al., 2014), the Behavioral Change Wheel, or the Theory of Planned Behaviour (Jenner et al., 2005; Clayton & Griffiths, 2008). Such theories acknowledge a range of influences on health behaviors although they also consider external communication cues such as messaging strategies. The Health Belief Model (Ghanbari, 2014) includes the role that “action to cues” play in HH decisions, namely that messages and reminders can shift attitudes. The Behaviour Change Wheel includes “persuasion” and “education” to highlight the influence that messaging/information may have on health behavior. While HH reminder messages play a role, there are a range of beliefs, such as perceived risk, self-efficacy, and susceptibility to social norms (Ghanbari, 2014) that can have an impact on the efficacy of any HH campaign. This study acknowledges the complexity of influences that affect health behaviours but also highlights the importance of understanding people s attitudes towards ‘action to cues’.

Shain and Kramer (2004) argue that the issue of health promotion at work is mainly divided between two concepts: that health behavior is an individual concern/responsibility (e.g., one worker habitually uses hand sanitizer while another doesn’t) and that health behavior can be influenced by the environment and thus is outside the control of the individual (e.g., workers want to sanitize their hands but they can’t locate sanitizer). In their study, the investigators call for the reduction of the individual/organizational conceptual divide. Workers’ involvement in health promotion initiatives can bridge this gap. Rather than health promotion design being something that is done *to* the worker, it is something done *with*, or even *by*, the worker.

As early as 2005, Jenner et al. (2005) highlighted the value of using the audience to select HH messages. Despite this, even clinical workers are rarely consulted in the development of HH messages. When they are, the consultation process is used primarily as a tool for message selection (Caris et al., 2018, Gaube et al., 2020, Judah et al., 2009). Consultation is often used within a wider methodology to scaffold and prime an intervention trial rather than to gain attitude knowledge to share in a wider academic setting.

Lambe et al. (2021), however, did conduct codesign tasks with stakeholders (including ranking and rating exercises) in a clinical setting and used findings to propose guidance for how HH initiatives can be constructed. Use of visual communication scored highly, and reprimands or coercive approaches were less favorably received. The research resulted in a toolkit to be used by other clinical institutions devising HH programs. We propose, by applying a consultative approach to office workers, that we will be able to uncover vital preferences and attitudes to inform HH initiatives beyond the clinical setting.

The qualitative views of users, while relatively rarely found in HH messaging research, can provide us with the “why” rather than the “what.” Thomas et al (2005) usefully used focus groups to establish more “human” approaches to messaging. They concluded that the process of using participant suggestions during implementation was invaluable. Our study presented here captures a large number of workers’ views that, too, should offer high value.

A neglected area of health communication research also relates to the style and visual approach of health message design. Images used in HH research tend to be “binary” in form, for example, pictorial or text-led rather than tested against stylistic variation. For instance, Rutter et al. (2021) presented alternative persuasive techniques but utilized photography only. More broadly, outside a health messaging context, pictorial style can affect the engagement of viewers (Kimle & Fiore,1992), and within a healthcare setting, that style can impact risk comprehension (Zikmund-Fisher et al., 2014). Our study hoped to uncover what preferences workers might have for image modes to underpin future design strategies.

Studies citing the importance of positive/negative bias of messages are more easily located (Jenner, 2005; Caris et al. 2018). Jenner (2005) argued that the conventional belief around health messaging (that gain framed messages are more effective) is challenged when applied to HH communication in a clinical setting: The benefit of HH is for the patient and not the clinician, who loses time through the activity. Since this study is focused on the workplace, it is arguable that gain framing that reduces risk of a worker themselves becoming ill—may be more applicable and relevant. Attitudes to framing was therefore a topic worthy of study here.

## Method

An online survey consisting of a mixture of 20 open and closed questions was devised (see Additional Data). The questionnaire first introduced the concept of a hand sanitizer station with a video screen and then used further questions to focus on nuanced preferences for messaging tone and emotional approach.

A literature review was conducted to identify a range of HH messaging principles and successful messages that had worked previously in other settings (see, for instance, Rutter et al., 2021; Gaube et al., 2020). These findings were used to populate rating and ranking-style questions. Messaging approaches such as disgust, social norms, knowledge acquisition, loss, and gain framed were presented, and participants were asked to rate the perceived effectiveness of these messages and submit a rationale for their choice. Additional questions that focused on the design and form of message strategies were devised. These included questions about picture style, picture content, tone, and emotion. These were designed to aid insights into preferences for the “feeling” of the messages, for example, should they be commanding, humorous, and so on. Such questions allowed us to build further on Thomas et al.’s (2005) work.

The MSForms-based survey was iteratively developed and piloted by five “typical” workers to ensure question appropriateness. Those involved in piloting the questionnaire were known to the researchers as employees working in the commercial sector. A convenience sampling strategy was then employed as human resources/site managers from six large retail and commercial organizations were approached to circulate the survey. The organizations approached were from a list of existing placement contacts of the host university and selected due to being sizable institutions with over 10,000 employees. A sample “invitation” email was sent to site managers who then circulated it, electronically, to their staff on behalf of the researchers.

The project was approved by the Ethical Approval Committee of the University of Leeds number FAHC 20-059. The survey was designed to allow anonymous responses with questions sensitively worded. Staff volunteered to take part with no provided incentive, and all consent and further information were provided at the start of the survey.

Five hundred and twenty responses were collected and analyzed. The response rate was unknown as emails were circulated on our behalf. Quantitative data were analyzed for statistical significance in SPSS 28 using chi-square tests. Qualitative responses were examined using an iterative thematic analysis approach in MS Word 1.8 by one lead researcher. A second researcher then rethemed the dataset to ensure intercoder reliability. Participant quotes were attributed to more than one theme if appropriate.

## Results and Analysis

The demographic characteristics of the participants were as follows. The gender ratio of participants was 64% female and 36% male. Most participants were under the age of 55 (87%) and 92% were native English speakers. The data therefore show a bias toward native English-speaking female workers. Data were collected in July 2021 over a 1-month period in the United Kingdom. Data represent the views of workers many of whom had returned to work environments in between the second and third waves of COVID-19 infections. Level of engagement with the survey was high with an average completion time of 23 min.

### Attitudes to Being Involved in the Design of HH Messages

Staff were asked whether they agreed/disagreed with the following statement: “As a member of staff, I should be consulted on the design/content of messages that promote HH at my workplace.” Only 20% of participants thought that workers should be involved in the design of health messages. Twice as many workers (44%) felt that it was not important that they be involved, with also a high level of neutrality reported (35%).

**Figure 1.**
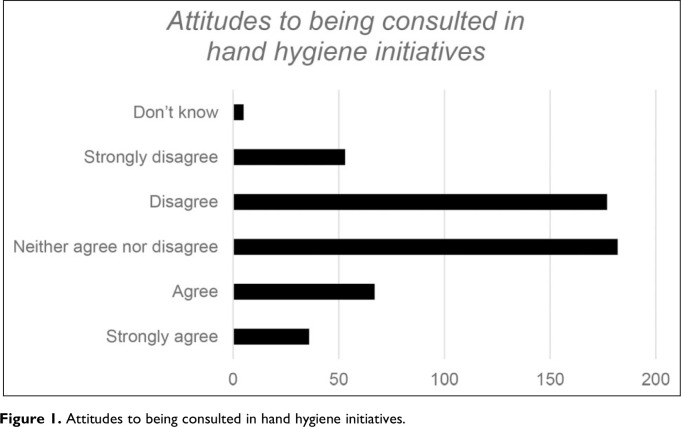
Attitudes to being consulted in hand hygiene initiatives.

There was a significant negative correlation between age and consultation (ρ = −0.10, *p* =.023), suggesting that older respondents were more likely to feel that they should be consulted.

### The Use of a Video Screen: Quantitative and Qualitative Results

**Figure 2.**
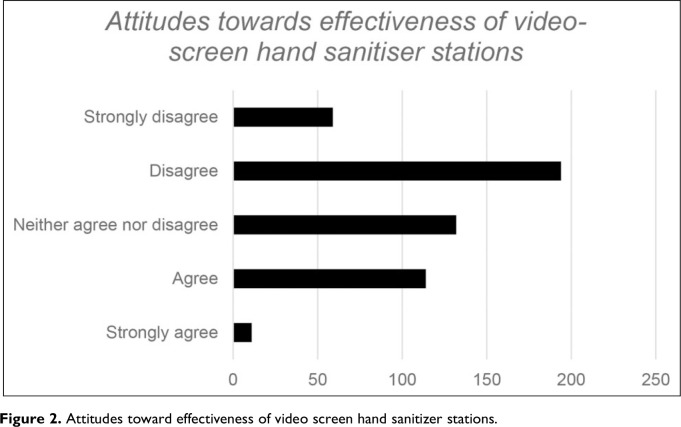
Attitudes toward effectiveness of video screen hand sanitizer stations.

There was some level of variance in terms of attitudes to the use of video screens for HH messaging. Figure 2 shows responses to the statement: “A video screen on a hand sanitizer dispenser would encourage more use of hand sanitizers in the workplace.”

Only a quarter of the participants (24%) expressed positive views towards the use of video hand sanitizers whilst twice as many (48%) responded negatively. Further, results of an open data field requesting a rationale for their answer demonstrated a degree of cynicism, a level of doubt and the dismissal of the use of video screens to improve the use of hand sanitizers. Almost half of the participants expressed a negative attitude toward the potential effectiveness of video screens. Many expressed, however, a particular belief that HH behavior is unlikely to be manipulated by any in situ intervention, not even a novel technology. More positive responses however mentioned the ability of a screen to attract attention and to act as a constant reminder of the need to use hand sanitizer.

Some participants reflected on the *type* of user the video screen would appeal to—such as more technology-focused users—as well as the general behavior of the workforce.

Moreover, these comments also reflected beliefs that were sometimes judgmental about others, such as “some people have to be told to do simple things,” “everyone does not sanitise,” and “everyone needs reminding. I can remember people not washing their hands after going to the toilet.” Such responses reflect the fact that workers do notice the HH of others. Table 1 lists emergent key themes from the data, in order of prevalence and with examples from transcripts.

**Table 1.** Negative Attitudes Toward the Use of Video Screens.

Negatively Positioned Themes	Examples of Coded Transcripts
No impact on HH practices	*“Those who want to sanitise will do so even if it has a video screen or not.”*
Static messaging is sufficient	*“I think the message is just as clear if it is written.”*
Skepticism over the use of the video itself	*“Why would a video screen make any difference?”*
People will ignore it	*“I really don’t think it would make any difference in my workplace - we are all intelligent adults and know the importance of hand hygiene. Those who are less hygienic (yes there are some) will not be swayed by a video or a company logo - they know what is right and choose to ignore it !!”*
Unnecessary wastage – expense/environment	*“It’s unnecessary expense. A conveniently positioned bottle works fine.”*

The main positive themes relating to the perceived effectiveness of video screens are shown in Table 2.

**Table 2.** Positive Attitudes Toward the Use of Video Screens.

Positively Positioned Themes	Examples of Coded Transcripts
Improve attention	*“Catches the eye more than static notices.”*
Ability to remind	*“I think it’s a good reminder to people of the current situation and why it’s important for people to regularly sanitise.”*
Ability to encourage	*“The novelty of a watchable screen may encourage people to stop and look, and in turn, use the hand sanitizer.”*
Ability to engage	*“We are living in a digital world now, I feel a video screen is more engaging.”*
Ability to be changed/updated	*“I think it would make people stop and read the display, especially if you change the message often.”*
Novelty	*“People respond to a gimmick/technology.”*
Ability to be interactive	*“It draws you in and feels more interactive.”*

### Message Selection

Participants were presented with a range of 10 statements to select from in terms of “the best statement to persuade you to use hand sanitizer at work” (see Table 3 for the full results).

**Table 3.** Preference Ratings for Message Selection.

Message	Message Type	Number (*n* = 520)
80% of common infections are spread by your hands	Knowledge Acquisition	*147 (28)*
There are more germs on your phone than toilet seat	Disgust	*90 (17)*
Clean hands keep loved ones safe	Consequence (gain framed)	*87* (17)
Thank you for cleaning your hands	Feedback (positive)	*63 (12)*
Only 50% of your team sanitized yesterday	Feedback (negative)	*44 (8)*
Clean germs off your hands or eat them later	Disgust (loss framed)	*36 (7)*
Dirty hands put your friends and family at risk	Consequence (loss framed)	*22 (4)*
Clean hands can reduce sick days by 21%	Knowledge acquisition	*21 (4)*
Are your hands as clean as your colleagues?	Social norms/surveillance	*6 (1)*
Your boss is watching you!	Social norms/surveillance	*4 (1)*

Examining the findings with respect to gender, the statement ‘80% of common infections are spread by your hands' was considered to be the most effective by 27% of men and 29% of women. However, there was a statistically significant difference between the second and third most popular choices (χ^2^ = 9.4, *p* = .009). Women (19%) were more likely than men (13%) to list Statement 6 (“There are more germs on your phone than on a toilet seat”) as the best, while 23% of men and 13% of women had Statement 4 (“Clean hands keep loved ones safe”) as their preferred choice.

Thematic analysis was conducted on the top four messages to discover principles for best practice and for issues of analysis efficiency. Since some statements contained several reasons, they could appear in several themes. “80% of common infections are spread by hands” was highly rated for a number of key reasons, highlighted here via thematic analysis in Table 4.

**Table 4.** Message Selection Thematic Analysis: “80%” Message.

“80% of Common Infections Are Spread by Hands”	
Theme	Example
Presents an impactful statistic	*“High percentage has an impact.”* “Factual content—high statistics.” “It’s impactful and the percentage is high!”
Presents new knowledge	“I was surprised at the statistic, which would encourage me more than a generic statement.” “I like numbers! It’s amazing that we don’t realise how much we touch with our hands.” “I think people may not know this information.”
Factual	“Because I don’t like threats or gimmicks. It’s factual and informative without being too personal.” “It doesn’t elicit an aggressive or guilty response, it is factual and clear without making me feel awkward about using it.” “It’s factual and doesn’t try to manipulate you though guilt or fear.”
Clear	“Straightforward.” “Makes more sense.” “Simple and to the point without being too negative.”

What is clear from this analysis is the need for the careful selection of impactful facts that are presented in a clear and non-manipulative way. The neutrality of this phrase was particularly valued. Some of the participants classed other messages as “guilt tripping,” “patronizing,” or “lecturing” but found this particular message more appropriate for a workforce.

Thematic analysis of reasons for the selection of “There are more germs on your mobile phone than on a toilet seat” produced the following results (Table 5).

**Table 5.** Message Selection Thematic Analysis: “Mobile Phone” Message.

Message: “There Are More Germs on Your Mobile Phone Than on a Toilet Seat”	
Theme	Example
Thought provoking	“It’s factually correct, funny and gets you thinking.” “Made me look at my phone in a different light.” “Most surprising.”
Relatable to the environment	“Because it directly links to the office environment and we are ‘hot desking’ so you could be using any desk equipment.” “It helps to highlight in real terms how dirty everyday objects get. Because I constantly use my keyboard and phone.” “Because my line of work is very office based and I am always using a keyboard or phone, this shocks me!”
Strong emotional impact	“I think the gross out method hits your emotional response more. It is a disgusting thought.” “I think it’s a statistic that most people would be really shocked by the ‘Ergh’factor.” “It sounds gross and is an awful thought so it makes you immediately want to wash/sanitise your hands.”
Simple	“It is informative and easy to understand.” “It was simple.” “Emotive and direct.”

As well as the factual aspect of this message being valued, the shock/disgust factor was also credited for its high rating. Of particular interest is the aspect of direct relevance that emerged from the justifications: that people constantly touch those particular objects was an effective factor in the messaging according to participants. In the construction of messages, everyday and relevant comparisons were positively received.

Thematic analysis of reasons why “Clean hands keep loved ones safe” was chosen is shown in Table 6.

**Table 6.** Message Selection Thematic Analysis: “Loved Ones” Message.

Message: “Clean Hands Keep Loved Ones Safe”	
Theme	Example
Incentivizing	“It’s a positive message and gives people an incentive to clean their hands.” “No one would want to make loved one ill.” “It makes to think more that your personal actions have a direct effect for those you care about most.”
Personal	“It does not mention work and so seems more interested in the persons safety.” “My loyalty to and love for my family far outstrips the company/colleagues/job.” “Because family and loved ones are more important to me than work.”
Relatable/applicable	“The reference to loved ones is emotive and makes you think of your parent/grandparents/more vulnerable people.” “I have clinically extremely vulnerable people in my family. I worry most about them.” “Makes you think of others close to you.”

This statement seems effective due mostly to the value placed on personal priorities. Participants appreciated the positive tone of this message alongside its relevance, encouraging people to think about their home lives and personal ramifications of HH. This message was effective due to its domestic setting and its positive framing.

The fourth most successful messaging strategy was “Thank you for cleaning your hands.” Thematic analysis is in Table 7.

**Table 7.** Message Selection Thematic Analysis: “Thank You” Message.

Message: “Thank You for Cleaning Your Hands”	
Theme	Example
Positive reinforcement	“Positive reinforcement, treat people like grown ups and make them feel good for doing the right thing rather than feel bad for not positive message rather than a fear message.” “I feel praise and encouragement work better than scare stories and veiled threats.” “Positivity is vital to encouragement.”
Rewarding	“Fits into gamification that there is some element of recognition/praise.” “Fun personalized message, you only get it when you’ve used the machine.” “It’s a nice message to receive.”
Simple	“Simple.” “Just a simple thank you is enough.” “Quick and to the point, says exactly what has been done in a direct manner.”

Like the other messages, simplicity was highly valued: Direct and clear statements were well received by participants. Positivity and reward were also of particular value with participants extolling the virtues of positive motivating statements over negative ones. The popularity of this message also reflects the value of the positively framed “Clean hands keep loved ones safe” (17%) above the “Dirty hands puts your friends and family at risk” (4%) approach. Within the rationale data, participants also expressed qualities they didn’t value, such as messages being “patronizing,” “lecturing,” “guilt tripping,” or “threatening.”

### Message Style

Table 8 lists responses to the multianswer question “Which style of message would be most effective in encouraging you to use hand sanitizer at work?” Some participants gave more than ONE response.

**Table 8.** Message Visual Style Selection (Multiple Options Allowed).

Style of Visual Message	Frequency
Scientific photographic image	161
Serious illustration	129
Fun cartoon	119
It does not matter	118
Text only	109
Everyday photographic image	96
I do not know	38
Other	22

A preference emerged for use of photographic or serious illustration over cartoon imagery or, say, text only. This was particularly true for men, who were significantly more likely than women (χ2 = 9.20, *p* = .010) to consider serious illustration to be the most effective style of message.

Interestingly, the fourth most popular view was that it doesn’t matter, highlighting an apathetic attitude to messaging form and highlighting a view that the *appearance* of a message has no impact on its effectiveness.

### Picture Content

**Figure 3.**
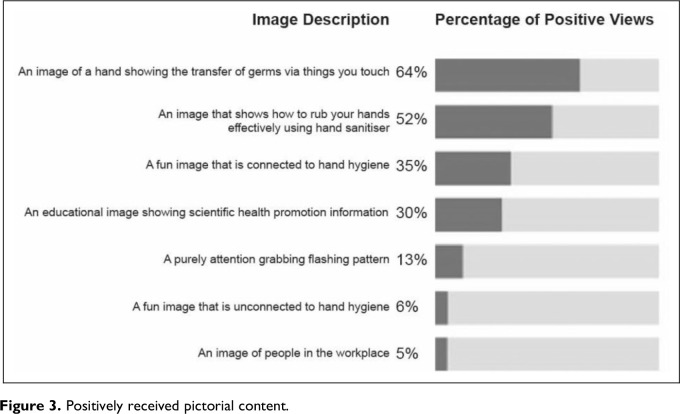
Positively received pictorial content.

**Figure 4.**
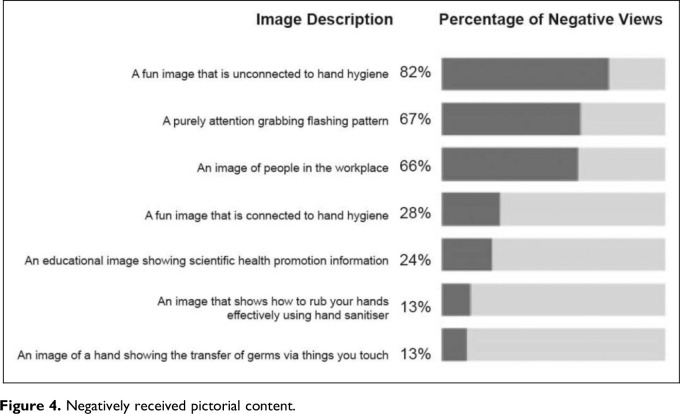
Negatively received pictorial content.

Participants were asked to rate each pictorial content according to their perceived effectiveness. Pictorial content with a positive rating (either “good” or “very good” rating) are shown in Figure 3. Pictorial content with a negative rating (either “poor” or “very poor” rating) are shown in Figure 4.

The results reveal preferences for educational or instructional images of hands connected to HH. Less popular choices include abstract approaches (e.g., a flashing pattern), images of people in the workplace and images unconnected with HH. Relevancy seems more important than decorative or purely attention-seeking imagery.

### Message Tone

Participants were asked to select message tones they considered effective. They were able to select more than one answer, as shown in Table 9.

**Table 9.** Preferences for Message Tone.

Tone of Message	Frequency
Positive—highlighting benefits	331
Trustworthy/educational	239
Friendly/supportive	206
Funny/informal	111
Negative/warning of risk	99
Strong/commanding	76
Neutral	27
It does not matter	18
I do not know	5
Other	2

There was a clear preference for positively toned messages, with benefit-led, trustworthy, and friendly messages scoring well. Risk-led, commanding, and neutral images were classed as less effective overall. This echoes earlier results related to message selection where there was a clear preference toward the need for positivity. These particular results also corroborate the value of more educational and trustworthy approaches evident, for example, in the use of scientific or serious illustration. In addition, 209 further responses via a reason field within the survey provide further detail. A bias toward the positive is evident in many reasons. A typical response is shown in the comment: “I’d rather it come from a positive spin as opposed to being ‘shamed’ into doing it.”

Broader themes from the rationale text for the “positive” selection are presented in Table 10.

**Table 10.** Rationale for “Positive” Responses From Open Field.

Theme	Example
Protects staff morale/mood	“Although we understand it’s for the greater good, strong, commanding, negative, risk-reminding messages do little for morale.” “An amusing message would draw attention and lift people’s mood at the same time.” “You don’t want to feel attacked as soon as you step foot into the office, it should be a positive thing to sanitise your hands.” “Build people up, don’t tear them down. There’s too much negativity in society and the news today. Don’t add to it.”
Encourages trust	“Fear messages seem childish. Positive messages give employees respect and trust in the workplace.” “You won’t shame people into action. Trust them to do the right thing, and support/thank them for being trustworthy.”
Encourages discussion	“I think if they fun and positive people will want to see the next message is, it might start conversations in the office and thus others will want to do it as well to be part of the conversation.” “Getting the message across to everyone and people respond both to positivity and to humour and they talk about funny things with others.”
Encourages self-empowerment	“Do not want to feel like it is being done because someone in charge is saying so but that we are doing it for the benefits to ourselves & others.”

Further survey comments make clear how creatively challenging designing an appropriate message actually is: How can a serious topic be expressed both in an accessible and appealing manner?

## Discussion

The results above raise several issues worthy of further discussion. Only 20% of participants thought that workers should be involved in the design of health messages despite the apparent value of stakeholder input being evidenced in the literature (Jenner et al., 2005; Lambe et al.,2021). These findings suggest that there may be a recruitment challenge for any researchers/organization wishing to involve members of staff in the design of HH interventions. The findings also indicate that older respondents are more likely to engage with such an activity. While we were unable to locate other studies that identify older member of the workforce as more willing to take part in consultations (more generally or in relation to HH), this finding broadly aligns with studies that highlight the potentially proactive and adaptable nature of the older worker (Ng & Law, 2014). Potential levels of stress, in part due to COVID-19, may also play a role in work priorities (Saleem, Malik, & Qureshi, 2021). H&S interventions or initiatives may be seen as the responsibilities of human resources or H&S managers rather than the general work force. While the evidence from Pittet et al. (2000) highlights a positive outcome from staff involvement, the results suggest how staff initially might need a good reason to “buy-in” to any participation.

Overall, there were misgivings about the role that video could play in improving HH. The majority of negative comments were related to the view that hand sanitization occurs and *should* occur regardless of technological or messaging interventions, and a skepticism was evident in many answers. In terms of models of behavior change such as the Health Belief Model, some workers tended to believe that internal “health motivation” would dominate and disarm “cues to action”: No messages would make a difference if the person habitually didn’t use hand sanitizer. Other views expressed a more positive response suggesting that if video screens are to aid HH, their content should be designed to show a variety of messages and employ movement to increase visual attention.

While video screens have been used in previous HH projects (Judah et al, 2009) to allow for easy message alternations, few studies exploit the moving image capability of the video screen despite its wide spread adoption in, say the advertising industry. McQuire (2011) points out that while having the potential to attract/distract the passer-by, video screens often have “narrowly” focused and limited programming and thus need careful planning to succeed as a communication tool. Given the use of video technology in hand sanitization stations is relatively new, respondents perhaps have limited experience or have not hitherto considered its use.

From the survey, it is shown that workers are sensitive to wastage—both of money and energy—and so interventions have to be shown to be cost effective for the company and environment. The video screens proposed actually have low wattage and energy-saving capabilities but are still more energy draining than mechanical sanitization devices. In order to get the buy-in from workers, new devices should be introduced carefully with energy impact communicated clearly alongside the key benefits of their usage for workers.

Of particular note in terms of message selection was the lack of popularity for the authority/surveillance messages such as “Your boss is watching you.” While studies have found that images of authority/surveillance were effective (Beyfus et al., 2016) in raising HH levels, these results show that a more positive and educational approach may work better in a more general workplace environment, in concurrence with Lambe et al. (2021). Women were more likely than men to think that disgust messages such as “Clean germs off your hands or eat them later” would be effective. This contradicts other studies where disgust had a larger effect on men than women (Judah et al., 2009). The gender bias in our study may be an attributing factor in this result but is worthy of further research.

Results across the whole survey highlight the preference for the positive, both in terms of message tone and message selection. This supports the notion that positive framing remains a valued tactic for positioning HH messages (Jenner, 2005) even when applied to a commercial work setting. Positively positioned messages about loved ones’ safety or the use of an immediate “thank you” were valued by participants. The triggering of disgust, as discussed at length by Curtis (2013), may also be an effective HH strategy when applied in the workplace. Interestingly, that workers connected HH messages overall to issues of morale or trust suggests that even relatively small messages within an environment may create larger impressions of the organization. Overall, messages that were positive, factual, relatable, and simple were more valued than others.

In terms of visual style, a preference for photographic and serious illustration emerged though the fourth most popular view was that style didn’t matter. This apathy also is reflective of the lacuna of research about picture/message style and the impact it has on behavior. That images were classed as more effective than words alone guide us toward a deeper consideration of the role of the image in HH messaging design and in particular the use of more factual style images, echoing the popularity of the factual content of “80% of infections” message. This would need additional consideration in the light of the other highly selected messages such as “keep your loved ones safe” or “there are more germs on your mobile phone than on a toilet seat” where more everyday images could be better suited. These findings suggest that serious illustrations may be more effective than others, but further research is needed.

The tone of HH written messages seemed to matter much more to participants than visual style. In total, 118 participants thought visual approaches didn’t matter as opposed to only 18 participants who considered written tone unimportant. Interestingly, visual style and tone are related, for example, a “fun” image is likely to be positive in tone. This wasn’t considered overall, in the data from participants. Trying to combine scientific imagery with positive message framing would, for instance, be a creative challenge. There remains much work to be done on the effect of visual style on health messaging more generally. These results illustrate a sense of indifference or lack of sensitivity within the audience and indeed among other researchers, at least consciously, regarding image diversity and its potential effect.

There was a strong preference for relevant image content, for example, hand images about HH or messages about relevant items such as mobile phones. This finding reflects Lang’s (2006) mediated messages model that foregrounds *relevancy* as a strong criterion for message encoding and retention in a health context. We can consider further how we might introduce *novelty*, another key criterion within Lang’s (2006) model, into image strategies. In our study, well-rated messages provoked thought or surprise also suggesting that novelty can play an important role. Movement, interaction, or bespoke and dynamic messaging might be other ways novelty can be achieved, and this requires further research.

There are a number of limitations with this study. Given that this survey was speculative, for example, not showing actual videos in situ or testing their effect with users, the results cannot capture an exact sense of the effectiveness of the video messaging technology nor the actual effectiveness of messaging strategies. What the results do provide more clearly is a snapshot of a workforce’s sense of the value of HH messaging 18 months after the beginning of COVID-19, and their broader sense of what HH interventions should look and feel like. Another shortcoming of the survey arose because the employers who assisted us in circulating the survey have a predominantly female workforce. Convenience sampling prevented control over the balance of participants and the population distribution cannot easily be described. The statistical analysis indicated that the sex of respondents may have had an impact on message preferences, so this is an area that would justify further investigation. The study would also have benefited from taking a longitudinal stance, measuring perceptions pre- and post-COVID-19 to understand how/if perceptions shift over time. This study has a UK focus and as such is bound by national cultural norms—a further comparative study would enable us to see how culturally specific these responses were.

## Conclusion and Recommendations

The study contributes new knowledge in several aspects as it is the first study of its kind to study the views of a commercial workforce on approaches to HH messaging. First, it highlights how new hand sanitizer technologies require careful introduction, considering workforce perceptions around wastage and overall communication value. Second, this study has shown a strong preference within the workforce for positive approaches to HH messaging. The use of visual messages that motivate via positive reinforcement and via educational and trustworthy approaches are perceived as effective strategies. We have highlighted that tone of written language mattered more to participants than image style though photographic and serious illustrations were classed as more effective than cartoons.

The use of open and qualitative methodologies to capture the views of those whose hygiene behavior we seek to improve has been shown to be of value in highlighting areas of cynicism and doubt as well as potential strategies to consider. We believe that a catalyst for change may come from similar qualitative methodologies at the very beginning of the design process. Our findings are important as they can shape the creation of future HH campaigns or HH interventions for the workplace and provide a starting point for creative thinking in this area.

## Implications for Practice

- Organizations should inform workers of economic and environmental cost of health interventions in relation to their benefits, to waylay cynicism.
- HH intervention trials in the workplace should prioritize messages that are positive, factual, relatable, and simple.
- HH messaging interventions in the workplace should include relevant imagery where possible.
